# Aberrant Expression of Interleukin-1β and Inflammasome Activation in Human Malignant Gliomas

**DOI:** 10.1371/journal.pone.0103432

**Published:** 2014-07-23

**Authors:** Leonid Tarassishin, Diana Casper, Sunhee C. Lee

**Affiliations:** 1 Department of Pathology (Neuropathology), Albert Einstein College of Medicine and Montefiore Medical Center, Bronx, New York, United States of America; 2 Department of Neurosurgery, Albert Einstein College of Medicine and Montefiore Medical Center, Bronx, New York, United States of America; Swedish Medical Center, United States of America

## Abstract

**Objective:**

Glioblastoma is the most frequent and malignant form of primary brain tumor with grave prognosis. Mounting evidence supports that chronic inflammation (such as chronic overactivation of IL-1 system) is a crucial event in carcinogenesis and tumor progression. IL-1 also is an important cytokine with species-dependent regulations and roles in CNS cell activation. While much attention is paid to specific anti-tumor immunity, little is known about the role of chronic inflammation/innate immunity in glioma pathogenesis. In this study, we examined whether human astrocytic cells (including malignant gliomas) can produce IL-1 and its role in glioma progression.

**Methods:**

We used a combination of cell culture, real-time PCR, ELISA, western blot, immunocytochemistry, siRNA and plasmid transfection, micro-RNA analysis, angiogenesis (tube formation) assay, and neurotoxicity assay.

**Results:**

Glioblastoma cells produced large quantities of IL-1 when activated, resembling macrophages/microglia. The activation signal was provided by IL-1 but not the pathogenic components LPS or poly IC. Glioblastoma cells were highly sensitive to IL-1 stimulation, suggesting its relevance *in vivo*. In human astrocytes, IL-1β mRNA was not translated to protein. Plasmid transfection also failed to produce IL-1 protein, suggesting active repression. Suppression of microRNAs that can target IL-1α/β did not induce IL-1 protein. Glioblastoma IL-1β processing occurred by the NLRP3 inflammasome, and ATP and nigericin increased IL-1β processing by upregulating NLRP3 expression, similar to macrophages. RNAi of annexin A2, a protein strongly implicated in glioma progression, prevented IL-1 induction, demonstrating its new role in innate immune activation. IL-1 also activated Stat3, a transcription factor crucial in glioma progression. IL-1 activated glioblastoma-conditioned media enhanced angiogenesis and neurotoxicity.

**Conclusions:**

Our results demonstrate unique, species-dependent immune activation mechanisms involving human astrocytes and astrogliomas. Specifically, the ability to produce IL-1 by glioblastoma cells may confer them a mesenchymal phenotype including increased migratory capacity, unique gene signature and proinflammatory signaling.

## Introduction

Glioblastoma multiforme (GBM) is the incurable highly malignant tumor of the brain with poor prognosis. Average survival following standard therapy (surgery, radio and chemotherapy) is 15 to 18 months [1]. Additional adjuvant therapies aiming at angiogenesis and tumor immunity are currently being investigated [2]–[6]. While most glioma studies are focused on specific anti-tumor immunity, little efforts are being directed at investigating the role of innate immunity. Innate immunity is a non-specific host response to pathogens or endogenous danger signals (tumor) that typically involves the generation of cytokines, chemokines and other inflammatory mediators that are important in the outcome as well as establishment of effective adaptive immunity [7]–[10]. Recent studies also emphasize the importance of tumor microenvironment (including glial and neuronal elements) in glioma progression and the role of glioma cells themselves as the producers and targets of inflammatory mediators [11]–[14]. Mounting evidence supports that chronic inflammation is a crucial event in carcinogenesis and tumor progression [13], [15]–[17]. For example, chronic overactivation of the IL-1β system has been considered a tumor promoting condition, arguing in favor of IL-1β inhibition for tumor prevention or therapy [15], [17].

While investigating the role of IL-1 in the CNS innate immune responses, we came across several intriguing observations that strongly implicate IL-1 as a tumor promoting agent in malignant glioma [14], [18]–[23]. IL-1 is the strongest inducer of pro-angiogenesis and pro-invasion factors such as VEGF and MMPs in human astrocytes and glioma cells. A number of lesser known pro-tumor genes such as IGF2 [24] are potently upregulated by IL-1 in astrocytes [25]. IL-1 is the top inducer of astrocyte/glioma miR-155 [26], [27], a microRNA implicated in inflammation-induced cancer formation [28], [29] and the most differentially upregulated in GBM (vs. malignant oligodendrogliomas) [30]. miR-155 targets suppressor of cytokine signaling (SOCS) proteins potentially leading to overactive Stat3, a transcription factor important in glioma progression [31]–[34]. Our recent GBM secretome study also revealed that IL-1 upregulates secretory molecules implicated in glioma progression such as MMP2, tenascin-C, galectin-1, pentraxin 3, IL-8 and MCP-1, while (down)modulating numerous extracellular matrix (ECM) and ECM-modulating proteins [14]. These results together suggest that IL-1 controls crucial aspects of glioma signaling and progression. Another aspect that is adversely impacted by IL-1 is anti-tumor immunity. IL-1 has been shown to suppress antitumor immunity and vaccine efficacy through expansion of myeloid-derived suppressor cells (MDSC) and generation and expansion of Th17 cells which activates Stat3 in tumor cells [35], [36]. Certain chemotherapeutic agents also trigger IL-1β release through lysosome destabilization and cathepsin B release, which in turn, curtails anticancer immunity [36].

In the current study we report aberrant expression of IL-1 proteins by human GBM cells, especially following stimulation with IL-1 itself. This finding is highly relevant given the inability of *human* astrocytes to generate IL-1. IL-1 is the strongest known activator of *human* astrocytes and *human* glioma cells, suggesting that aberrant innate immune interactions involving IL-1 could have significant impact on glioma progression and the integrity of CNS tissue.

## Materials and Methods

### Cells

Glioblastoma cell lines U251 and U87 (HTB-14) originally obtained from American Type Culture Collection (ATCC) were cultivated in high glucose (4.5 g/L, Catalogue # MT-10-013-CV, Corning) DMEM with 10% fetal bovine serum (FBS) and a mixture of antibiotics-antimycotic “Anti-Anti” (Life Technologies) (“complete medium”). Patient-derived glioma cell lines were obtained from Department of Neurosurgery, Montefiore Medical Center, Bronx NY. TJ14 was from a 7 year old female with astrocytoma, LL72 (GBM2) was from a 61 year old male with glioblastoma, LB1012 (GBM1) was from a 72 year old male with glioblastoma. Collection of fresh tumor specimens from patients with primary gliomas was approved by the Montefiore Medical Center Institutional Review Board as previously published [37]. Cells were maintained in RPMI 1640 (10-040-CV, Corning) with 10% FBS and Anti-Anti mix. Cells were plated at 1×10^4^ cells per well in 96 well plates for ELISA and immunostaining and at 1×10^6^ cells in 6 cm dishes for real-time PCR and western blot.

Human umbilical vein endothelial cells (HUVEC-2) (BD Biosciences) were grown in Cascade Biologics’ Medium 200 (M200) with Low Serum Growth Supplement (LSGS) (Life Technologies/GIBCO/Invitrogen) in 10 cm dishes (BD Biosciences) coated with 0.1% gelatin (Sigma-Aldrich) until cells reached 80–90% confluence. Cells were discarded after 5 passages. HEK293 cells were grown in complete medium, as described above.

### Preparation of human fetal astrocyte and microglial cultures

Human fetal astrocytes cultures were prepared as previously described [26], [38], [39] and according to the protocols approved by the Albert Einstein College of Medicine Institutional Review Board. Briefly, brain tissues of abortuses were dissociated by mincing and trituration and incubated in 0.05% Trypsin-EDTA for 45 min at 37°C. This was followed by filtering through 270 µM and 130 µM pore nylon meshes. Cells were seeded in complete media and cultured till monolayer was formed (∼2 weeks). Thereafter, monolayers were passaged ∼every 2 weeks at least 3 times ( = G3) to enrich for astrocytes (>99% GFAP+). Astrocytes were plated at 1×10^4^ cells per well in 96 well plates for ELISA and immunostaining and at 1×10^6^ cells in 6-cm dishes for real-time PCR and western blot. Microglial cultures were prepared by pooling the medium of monolayer cultures at 2–3 weeks in vitro, as previously described [38], [40]. Microglial cultures were >98% Iba-1+.

### Reagents and cell treatments

Human IL-1α, IL-1β, and IFNγ were purchased from Peprotech and used at 10 ng/ml unless indicated otherwise. IL-1α and IL-1β were used interchangeably with the same results. Human IL-1ra was purchased from Peprotech and was used at 1 µg/ml. Poly IC was purchased from Sigma and used at 10 µg/ml. LPS from *Escherichia coli* strain 0111:B4 was purchased from Sigma and was used at 100 ng/ml. Cells were treated for 6 h for Q-PCR and 24 h for ELISA, unless indicated otherwise. Cell treatment with inflammasome activators was performed following the published protocols [41]–[43]. ATP (adenosine 5′-triphosphate disodium salt) was purchased from Sigma and was used at 5 mM. ATP was added to cultures 30 min before cell harvest. Nigericin sodium salt was purchased from Sigma and was used at 20 µM. Nigericin was added to culture 1 h before cell harvest. Lactacystin was purchased from Santa Cruz Biotechnology and was added to culture 10 min prior to cell stimulation.

### Antibodies

Mouse anti-human IL-1β, 1∶250 (R&D Systems); rabbit anti-human NLRP3, 1∶250 (Sigma); rabbit anti-human ASC 1∶200 (Santa Cruz); goat anti-Annexin A2, 1∶200 (Santa Cruz); mouse anti-pSTAT3, 1∶250 (Cell Signaling), rabbit anti-STAT3, 1∶1,000 (Cell Signaling); mouse anti-β-actin, 1∶500 (Cell Signaling); anti-rabbit IgG conjugated with HRP, 1∶500 (ThermoScientific/Pierce); anti-mouse IgG conjugated with HRP, 1∶500 (ThermoScientific/Pierce); anti-goat IgG conjugated with HRP, 1∶5,000 (Rockland Immunochemicals); ImmPRESS polymer detection reagent (HRP conjugated anti-mouse IgG) (Vector Laboratories).

### Immunostaining

GBM cells were grown in 96-well plates (BD Biosciences) and fixed in ice-cold methanol for 30 min. Cells were then permeabilized by incubation with 0.3% Triton X-100 for 15 min followed by incubation with 3% H_2_O_2_ in order to block the endogenous peroxidase (30 min at room temperature {RT}). Then the cells were blocked with 10% normal goat serum for 1 h at RT and incubated with primary antibody (mouse anti-human IL-1β, dilution: 1∶100) for 1 h at RT followed by overnight incubation at 4°C. HRP-conjugated anti-mouse IgG (ImmPRESS reagent) was then added for 30 min at RT. Color was developed using diaminobenzidine (DAB).

### Real time RT-PCR

Quantitative real-time reverse transcription-PCR (Q-PCR) was performed as described previously [26], using porphobilinogen deaminase (PBDA) or glyceraldehyde 3-phosphate dehydrogenase (GAPDH) as endogenous controls. Briefly, total RNA was extracted with TRIzol (Invitrogen/Life Technologies), following the manufacturer’s instructions. PCR was performed using a SYBR green PCR mix and conducted with the ABI PRISM 7900HT FastPCR System (Applied Biosystems). All values were expressed relatively to endogenous controls. The median value of the replicates for each sample was calculated and expressed as the cycle threshold (*C_T_*; cycle number at which each PCR reaches a predetermined fluorescence threshold, set within the linear range of all reactions). Δ*C_T_* was calculated as *C_T_* of endogenous control gene (PBDA) minus *C_T_* of target gene in each sample. The relative amount of target gene expression in each sample was then calculated as 2^Δ*CT*^. Fold change was calculated by dividing the value (2^Δ*CT*^) of test sample by the value (2^Δ*CT*^) of control sample (control = 1). The primer sequences are previously published [39].

### Enzyme-linked immunosorbent assay (ELISA)

ELISA was performed using the DuoSet Kits from the R&D, as previously described [26], [44]. Briefly, polystyrene 96-well plates (Nunc) were pre-coated overnight at RT with specific capture antibody, then blocked with 1% BSA in PBS for 1 h at RT, and incubated with standard cytokine dilutions and cell cultures media (100 µl, undiluted) for 2 h at RT followed by washes with PBS plus 0.1% Tween 20, and incubation with biotinylated detection antibody for 2 h at RT. After the second wash the plates were incubated with HRP-streptavidin for 20 min at RT and washed again. The signal was developed after addition of 3,3′,5,5′-tetramethylbenzidine-peroxidase EIA kit (Bio-Rad) until color appeared and the reaction was stopped by 1 M H_2_SO_4_. Microplate reader (Dynex Technologies) was used to detect the signals with 450 nm and correction at 530 nm. The detection range of IL-1β ELISA was 3.9–250 pg/ml.

### Western blot analysis

Western blot analysis was performed as previously described [14], [26]. Briefly, cells in 6 cm dishes were scraped into ice-cold PBS, pelleted, lysed in buffer containing 1% Triton X-100 and 0.5% Tween 20 with protease inhibitors (Roche) for 30 min at 4°C and centrifuged at 10,000 rpm for 10 min. Thirty to fifty micrograms of protein was separated by SDS-PAGE in 4–20% gradient polyacrylamide gel and then transferred to polyvinylidene difluoride membrane (PVDF) membrane (Bio-Rad). The membranes were blocked in 5% nonfat milk for 1 h at RT, and then incubated with primary antibody overnight at 4°C followed the incubation with secondary antibody for 1 h at RT. Signals were developed using SuperSignal West Pico/Femto Chemiluminescence Substrate (ThermoScientific).

### Co-immunoprecipitation (Co-IP)

Cells were lyzed in PBS buffer containing 1% Triton X-100 and 0.5% Tween 20 with protease inhibitors (Roche) for 30 min at 4°C. After centrifugation at 10,000 rpm form 10 min the lysates were pre-cleared by incubation with Protein A Agarose (ThermoScientific) for 1 h at 4°C and after low speed centrifugation the supernatant containing 200 µg protein was incubated with 5 µg of mouse anti-human ASC monoclonal antibody (Santa Cruz) for 1 h at room temperature and Protein A Agarose (20 µl of 50% slurry) for 18 h at 4°C. At the next step the Agarose beads were washed 3–4 times with lysis buffer and proteins were eluted by boiling in Laemmli buffer and separated by SDS-PAGE in 4–20% gradient polyacrylamide gel (Bio-Rad). After electrophoresis the proteins were transferred to PVDF membrane, detected with rabbit anti-NLRP3 antibody and anti-rabbit IgG conjugated with HRP (ThermoScientific/Pierce).

### Transfection with IL-1β/GFP or GFP expression plasmids

The plasmid pEGFP-C3 encoding wild-type GFP optimized for brighter fluorescence and higher expression in mammalian cells was obtained from Clontech Laboratories, Mountain View, CA. The plasmid RG202079 in pCMV6-AC-GFP vector designed to express IL-1β in mammalian cells as a GFP-tagged protein was obtained from Origene (Rockville, MD). HEK293 cells and human astrocytes were transfected with pEGFP-C3 or RG202079 (IL-1B - GFP) plasmids in 96-well plate or 6 cm dishes using TransIT-TKO transfection reagent (Mirus BioLLC, Madison, WI) or Continuum™ transfection reagent (Gemini Bio-Products, West Sacramento, CA) following the manufacturer’s instructions. After 24 hr, GFP was detected in 96-well plate using Olympus fluorescence microscope. The cells from 6 cm dishes (after control for GFP expression by fluorescence) were scrapped into PBS buffer, lysed, and used for Western blotting.

### Application of anti-miR inhibitors and siRNA

Anti-miR-132, anti-miR-212 and control miR inhibitor (Negative Control #1) were purchased from Applied Biosystems. siRNA for human NLRP3 (Cryopirin) and control siRNA were obtained from Santa Cruz Biotechnology. siRNA for human annexin A2 and control siRNA were purchased from Thermo Scientific/Dharmacon. Transfection was performed with TransIT-TKO Transfection Reagents (Mirus BioLLC) as previously described [26].

### TaqMan real-time RT-PCR assay

All reagents including specific and control primers for TaqMan real-time RT-PCR were purchased from Applied Biosystems and the reactions were set according to the manufacturer’s protocol. Briefly, total RNA was purified by TRIzol (Life Technologies/Invitrogen). 10 ng of total RNA and RT primers for hsa-miR-132, hsa-miR-212 and the housekeeping gene RNU44 were used for reverse transcription (RT) with TaqMan MicroRNA Reverse Transcription Kit. Real-time PCR quantification was performed using TaqMan PCR primers and TaqMan Universal PCR Master Mix, No AmpErase UNG on the ABT PRISM 7900HT Fast PCR system (Applied Biosystems). Relative mRNA expression was determined as previously described [26].

### Preparation of GBM conditioned medium (CM)

GBM cell cultures were treated in different conditions (medium alone or treated with 10 ng/ml IL-1β, 1 µg/ml IL-1ra or both IL-1β and IL-1ra) for 15–20 min to induce cell activation. Cultures were then washed three times to remove input cytokines, followed by additional 22–24 h incubated in fresh medium to yield CM. The starting medium for the tube formation assay was M200 medium and for neurotoxicity assay - low-serum medium (DMEM+0.5% FBS).

### Tube formation assay

Assay was performed using “BD BioCoatTM Angiogenesis System-Endothelial Cell Tube Formation MatrigelTM Matrix 96-well plate” or in 96-well plates using BD MatrigelTM Matrix Basement Membrane. Briefly, BD Matrigel Matrix (∼10 mg/ml) was thawed overnight on ice at 4°C and added to the pre-cooled 96-well plates at 50 µl per well followed by incubation for 1 h at 37°C. HUVEC cells were trypsinized, pelleted by low speed centrifugation, and suspended in M200 medium or conditional medium (CM) from U87 cells, and then applied on Matrigel at 2–2.5×10^4^ cells/100 µl medium/well. The plate was incubated for 18 h at 37°C. After fixation with 4% paraformaldehyde for 15 min at RT, cells were visualized by phase-contrast microscopy. The number of tube-like structures with closed networks of vessel-like tubes was counted. The experiment was repeated at least 3 times.

### Neurotoxicity assay

The ability of GBM secretome to induce neuronal death was determined by exposing primary mixed human neuronal cultures to GBM conditioned medium prepared as described above. Neurotoxicity assay was performed by vital dye exclusion test 72 h later, as previously described [22], [25]. Controls consisted of treatment with medium alone (DMEM+0.5% FBS) or with recombinant IL-1 at 10 ng/ml. The results were scored by counting the number of dead (trypan blue positive) neurons in 4 different 200X microscopic fields per well, in 3 replicate wells.

### Statistical Analysis

**Figure 1:**
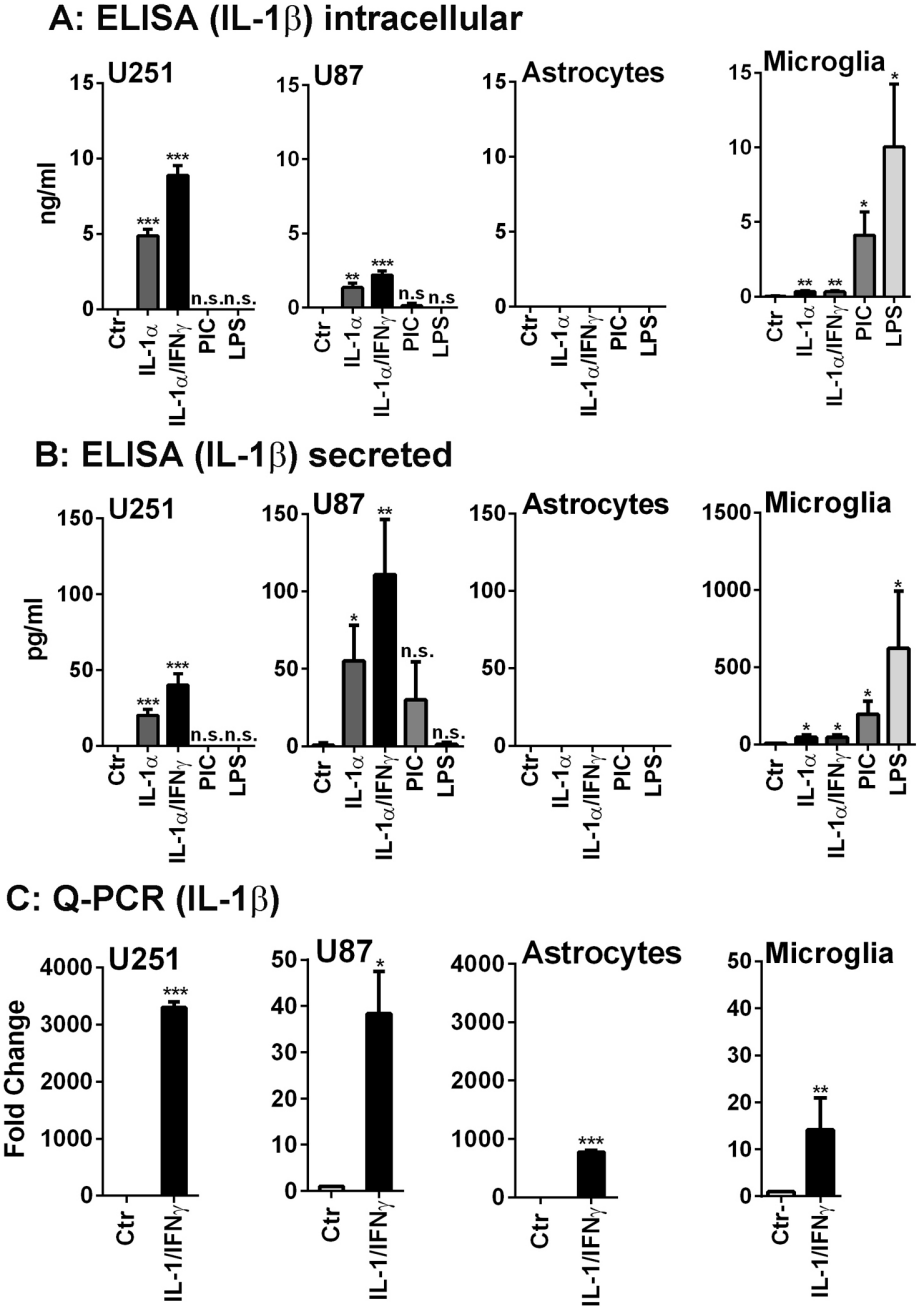
Human glioma cells produce IL-1 mRNA and proteins in response to IL-1 stimulation. The production of IL-1β protein (intracellular A and secreted B) was examined in U251, U87, human fetal astrocytes and microglia (from left to right) in response to IL-1α, IL-1α/IFNγ, poly IC (PIC) and LPS. The GBM cell lines (U87 and U251) produced IL-1β in response to IL-1α with IFNγ acting as a primer. Little or no IL-1 was induced by poly IC and none by LPS. Microglial IL-1 protein was induced by LPS >poly IC >> IL-1 (± IFNγ). Human astrocytes did not produce IL-1β protein. (C) By real-time PCR, all four cultures including human astrocytes expressed IL-1β mRNA in response to IL-1/IFNγ. Data shown are mean ± SD (n = 3). ***p<0.001, **p<0.01, *p<0.05, n.s. = not significant compared to control (Ctr). Also see Table S1.

**Figure 2:**
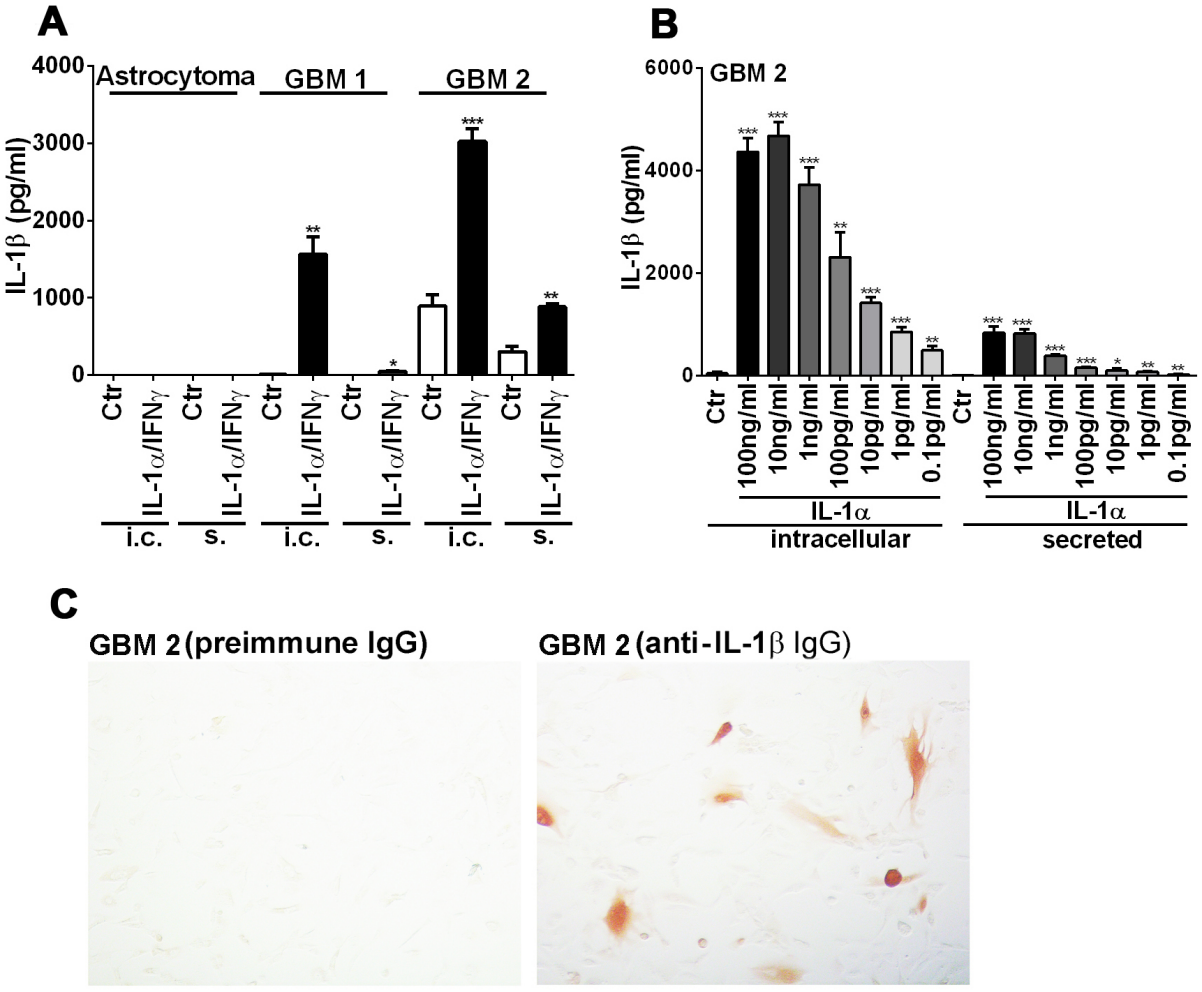
IL-1 expression in patient-derived glioblastoma cells. Three patient-derived glioma cell lines (one low grade astrocytoma and two GBM cells) were tested for IL-1 protein production spontaneously (Ctr) and following IL-1/IFNγ stimulation. ELISA was performed with cell lysates (intracellular = i.c.) and culture supernatants (secreted = s). (A) IL-1 protein production was seen in GBM cells only. In one of them (GBM2), IL-1 production occurred spontaneously (Ctr) and was potentiated by IL-1 (± IFNγ). A portion of IL-1 was secreted. (B) Dose-dependent induction of IL-1: production of IL-1β was examined in GBM2 cells after stimulation with different concentrations (0.1 pg/ml to 100 ng/ml) of IL-1α. (C) IL-1β immunostain of unstimulated GBM2 cells reveals scattered positive cells, while preimmune IgG (negative control) showed no positive cells. Data shown are mean ± SD (n = 3), ***p<0.001, **p<0.01, *p<0.05 vs. control (Ctr).

**Figure 3:**
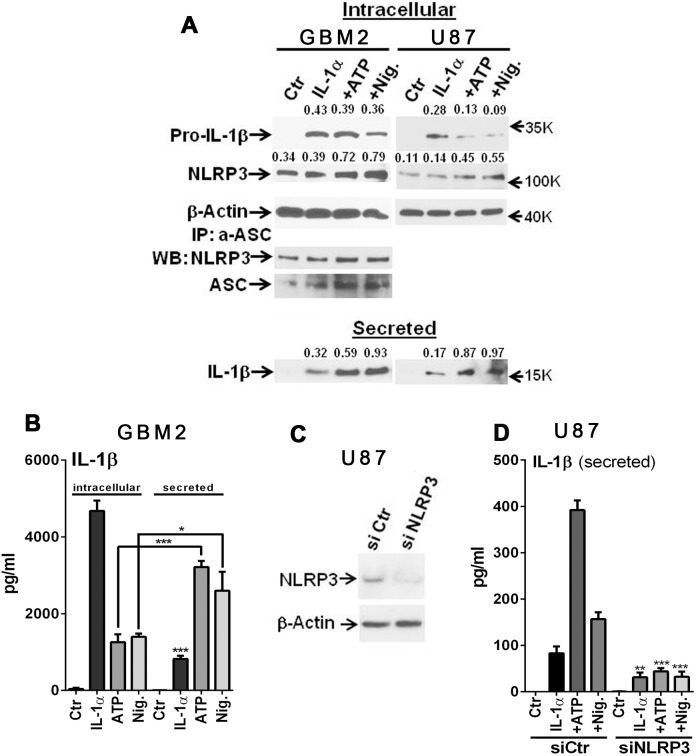
Inflammasome activation in GBM cells. (A) Western blot showing induction of intracellular 32 kDa pro-IL-1β and secreted 17 kDa IL-1β following IL-1 stimulation. Addition of ATP and nigericin (Nig.) increased IL-1β processing (decrease of proIL-1 and increase of secreted IL-1) in GBM2 and U87 cells. NLRP3 protein was detected in unstimulated (Ctr) cultures and showed a marginal increase after IL-1 stimulation. ATP and Nig substantially increased NLRP3 protein expression. NLRP3 protein was complexed with ASC (immunoprecipitation with anti-ASC antibody) in all conditions and the complex formation was increased by ATP and Nig. Numbers are densitometric ratios to β-actin for intracellular proteins and densitometry of IL-1β measured in concentrated culture supernatants (see Materials and Methods). Average densitometry data (NLRP3, pro-IL-1β and secreted IL-1β) from two experiments for both GBM2 and U87 are shown in Figure S1. (B) ELISA of GBM2 cells confirm enhanced IL-1β processing by ATP and nigericin. (C) Suppression of NLRP3 expression by siRNA in U87 cells. (D) NLRP3 siRNA-transfected U87 cells show significant reduction in the amount of secreted IL-1β in all three conditions (IL-1, + ATP, + nigericin) compared to control siRNA-transfected cells. Data shown are mean ± SD (n = 3) ***p<0.001, ** p<0.01, *p<0.05 by t-test.

**Figure 4:**
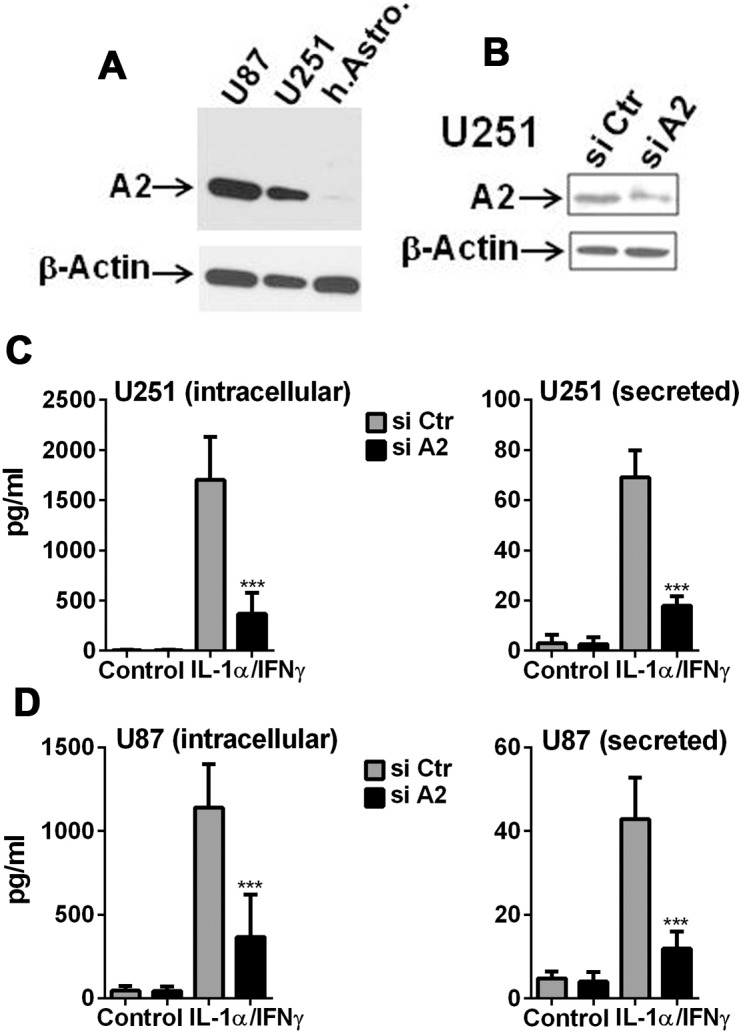
Annexin A2 promotes glioma IL-1 synthesis and release. The role of Annexin A2 in IL-1 production was examined by ELISA following transfection with A2-specific or control siRNA. (A) Western blot showing robust amounts of A2 protein expression in U87 and U251 compared to normal human astrocytes. (B) Western blot showing decreased A2 expression in U251 cells transfected with A2-specific siRNA vs. control siRNA. (C, D) The amount of IL-1β protein (intracellular and secreted) was determined by ELISA in U251 and U87 cultures following A2 knockdown. Data are mean ± SD (n = 3) *** p<0.001, ** p<0.01 (si Ctr vs. si A2), t-test.

**Figure 5:**
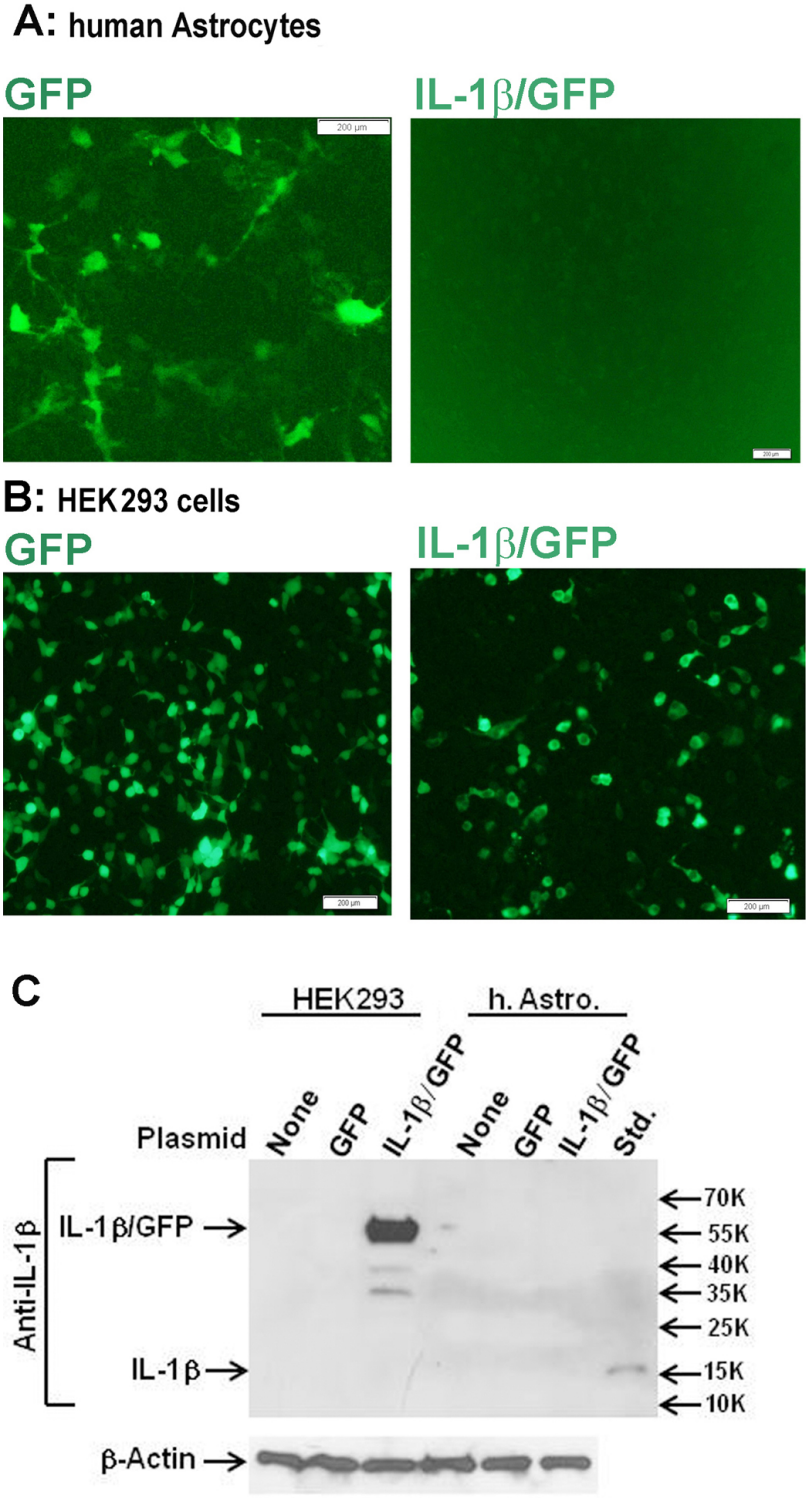
Human astrocyte IL-1β expression is actively suppressed. Human astrocytes or HEK293 cells were transfected with IL-1β/GFP or GFP expression plasmids as described in the Materials and Methods. (A, B) Fluorescence microscopy at 24 h after transfection reveals that control GFP vector but not IL-1/GFP vector-transfected astrocytes show green fluorescence. In contrast, HEK293 cells showed fluorescence in both conditions. (C) Western blot analysis of HEK293 cells (left) and human astrocytes (right) transfected with none (control), GFP plasmid (GFP), or IL-1 plasmid (IL-1/GFP). Incubation with anti-IL-1β antibody shows positive bands only in IL-1/GFP-transfected HEK cells and not in human astrocytes. The last lane (Std.) is a positive control (17 kDa recombinant IL-1β).

**Figure 6:**
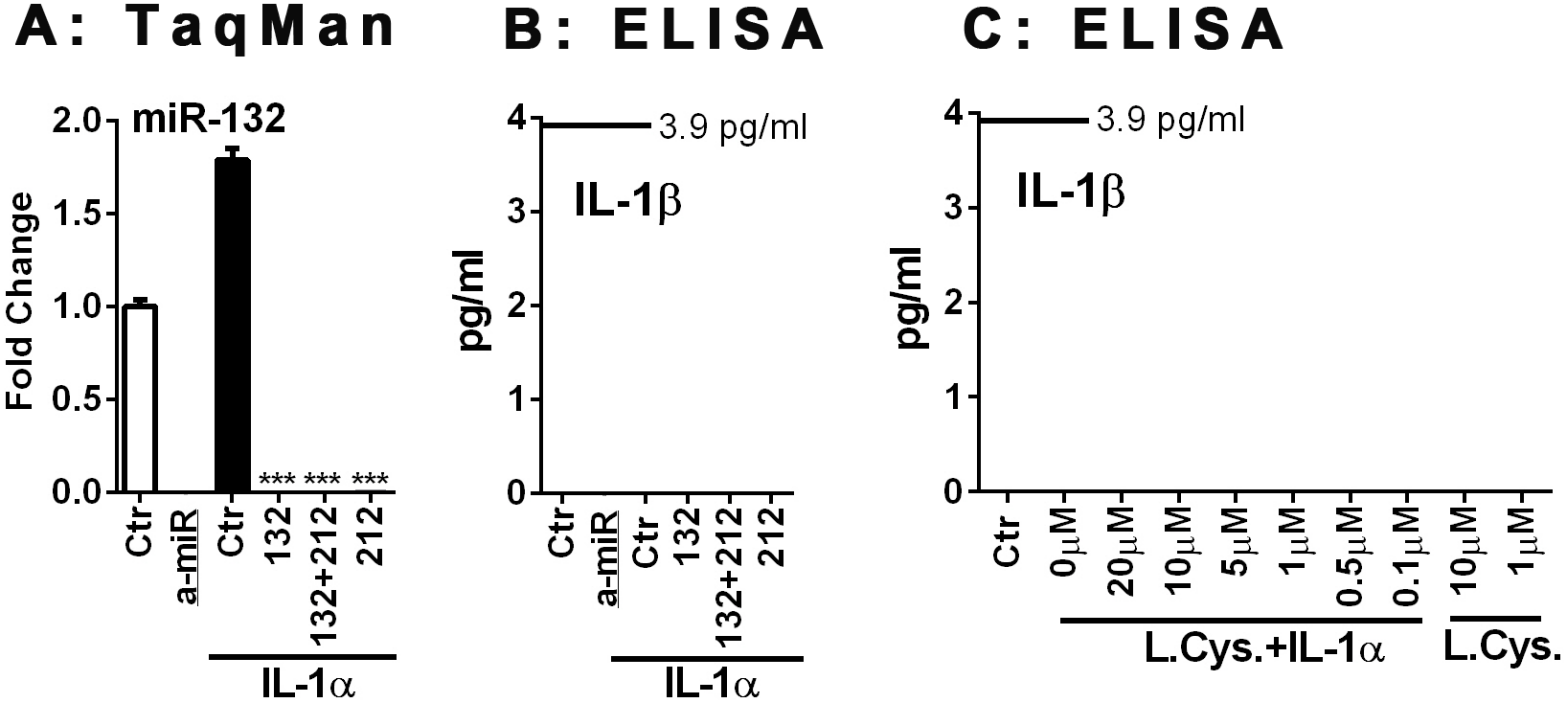
miR-132, miR-212 or the proteasome inhibitor lactacystin do not affect the expression of IL-1 in human astrocytes. Human astrocytes were transfected with specific or control anti-miR inhibitors (10 nM) for 48 h, and then stimulated with IL-1α for 24 h. (A) The expression of miR-132 was quantified by TaqMan real-time RT-PCR. Specific anti-miRs but not control anti-miR suppress miR-132 expression. (B) The culture supernatants were examined for the presence of IL-1β protein by sensitive ELISA with a lower detection limit of 3.9 pg/ml. There was no detectable IL-1β protein production in any of the human astrocyte cultures examined. (C) The effect of the proteasome inhibitor lactacystin on astrocyte IL-1β was examined. Astrocytes were treated with lactacystin at indicated concentrations with or without IL-1α, then cell lysates were subjected to ELISA after 24 h. IL-1β protein was undetectable under any conditions. Mean ± SD from triplicate cultures.

**Figure 7:**
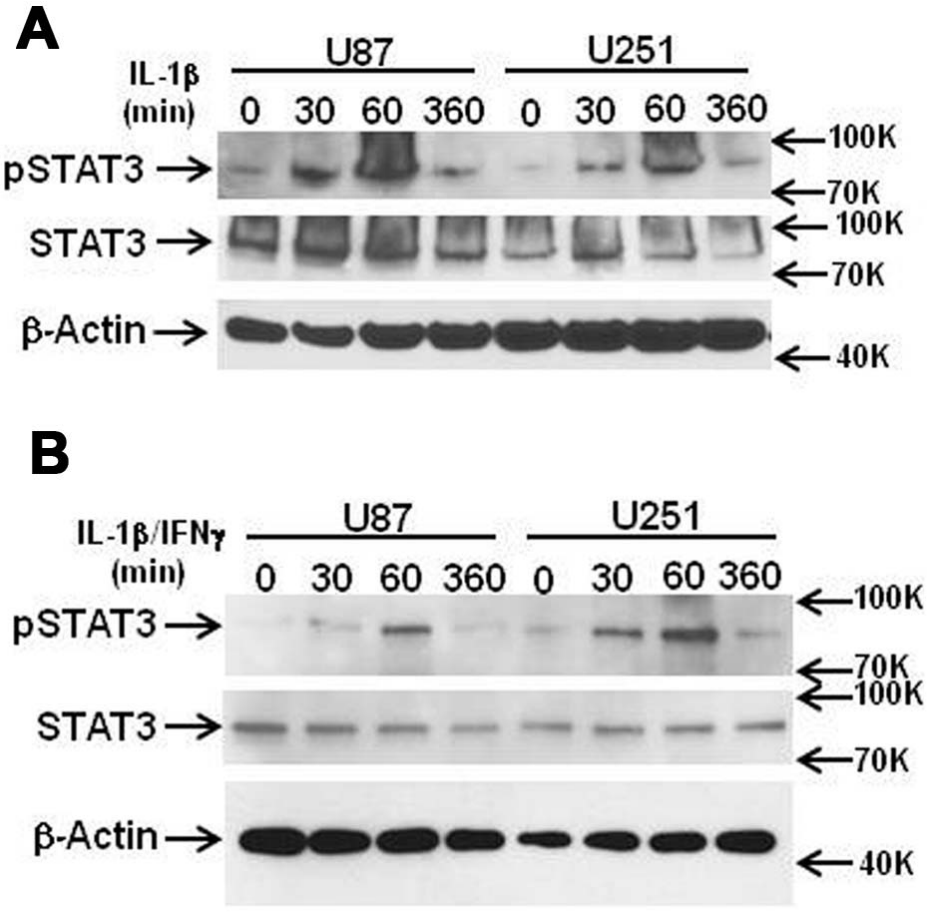
IL-1 (± IFNγ) activates Stat3 in gliomas. U87 and U251 cells were stimulated with IL-1 (A) or IL-1±IFNγ (B) and cells were harvested at indicated time points (min) for western blot. Blots were incubated with antibody to pStat3, total Stat3, as well as β-actin. Both cultures showed Stat3 phosphorylation reaching maximal at 60 min post IL-1 stimulation.

**Figure 8:**
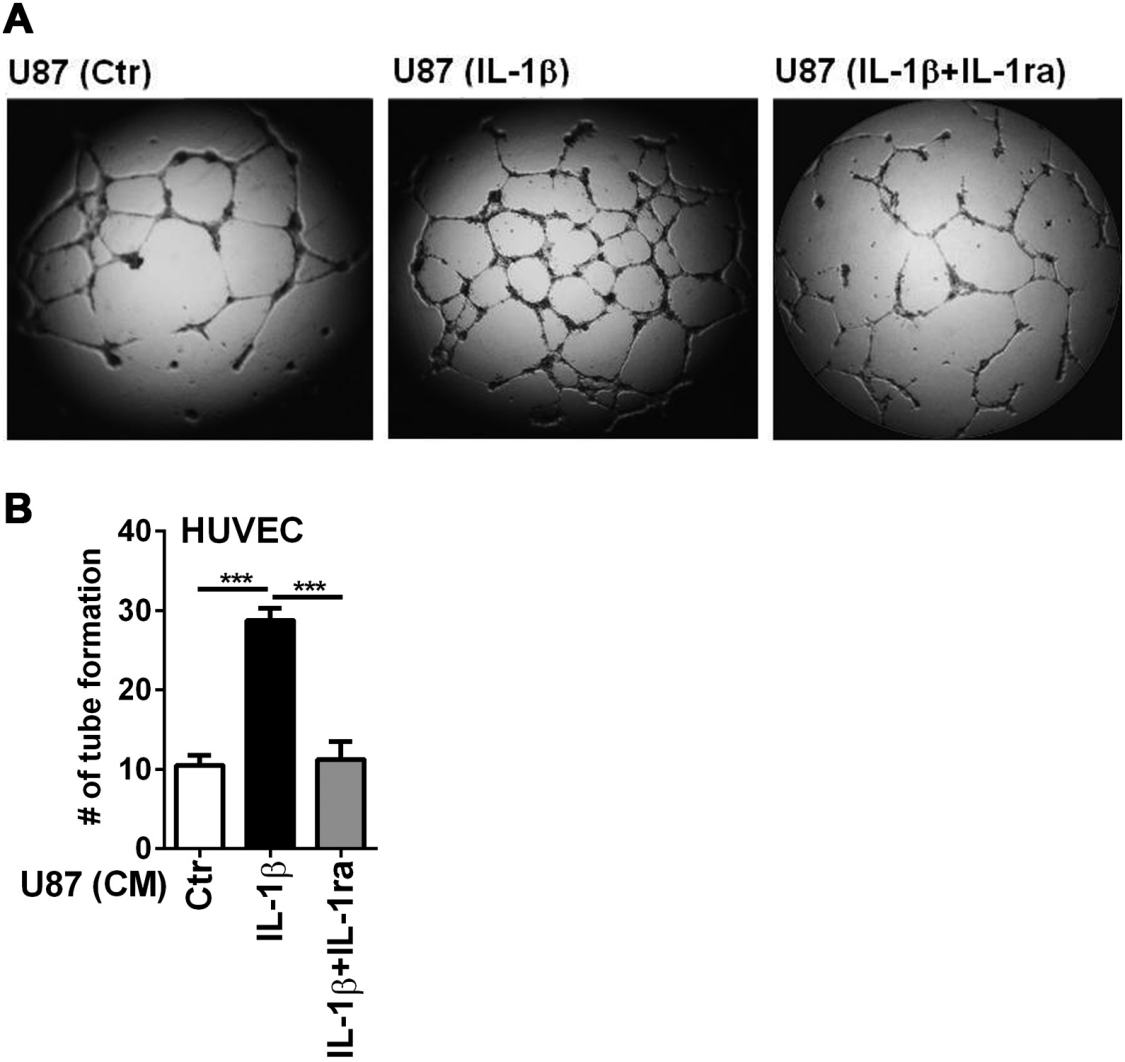
IL-1-activated U87 secretome promotes angiogenesis *in vitro*. Tube formation assay was performed using the BD BioCoat Angiogenesis System-Endothelial Cells Tube Formation Matrigel Matrix 96-well plate as described in the Materials and Methods. U87 conditioned media (CM) were prepared by incubating cultures with medium alone (Ctr), IL-1β (10 ng/ml), or IL-1β plus IL-1ra (1 µg/ml). HUVEC at 2.5×10^4^ cells were suspended in U87 CM and then added to the plates for 18 h at 37°C. Cells were then fixed in 4% PFA and viewed by phase-contrast microscopy. (A) Representative photography. (B) The number of closed network of vessel-like tubes was counted from three experiments. Data are mean ± SD ***p<0.001.

**Figure 9:**
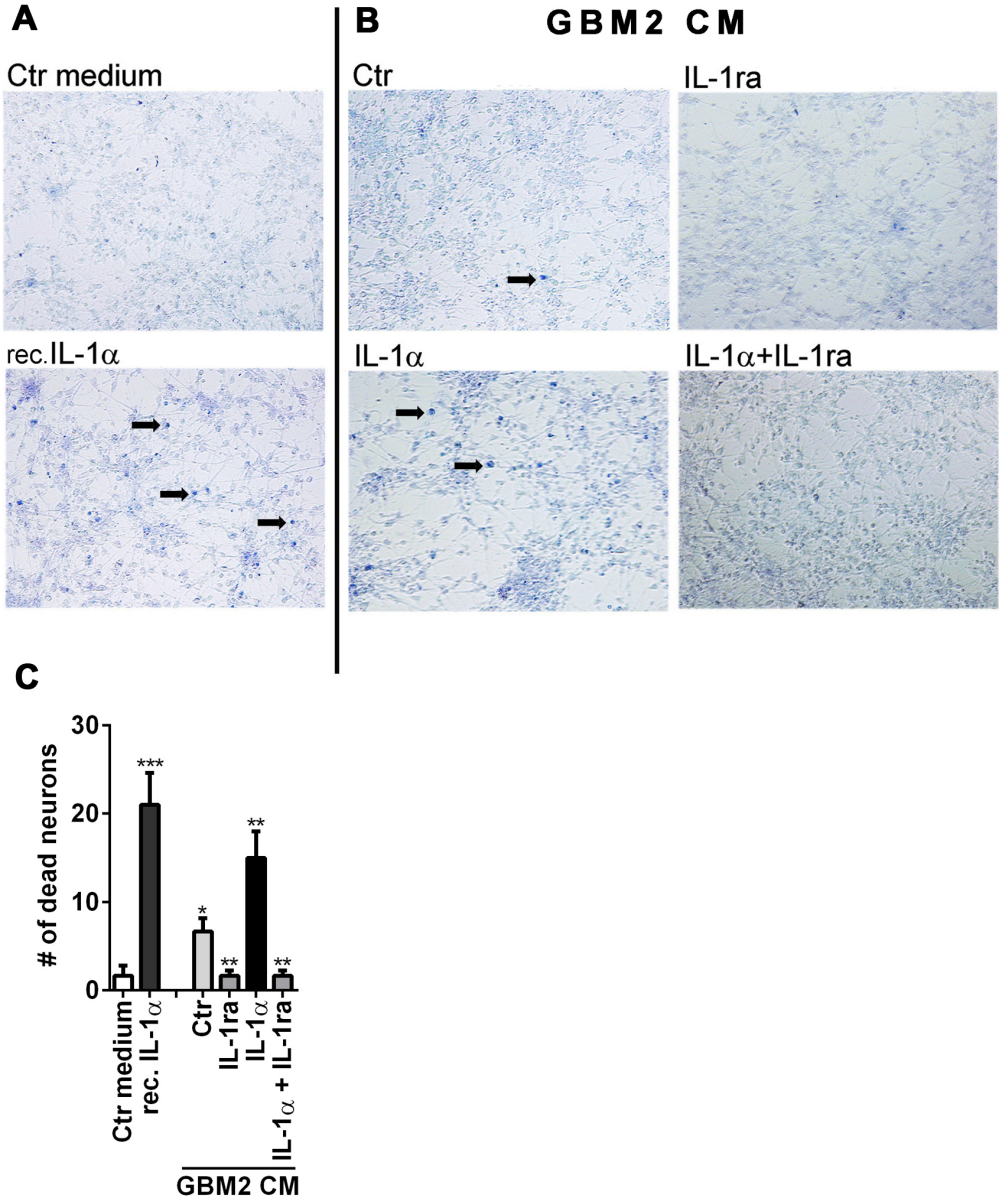
Glioma releases neurotoxic substances. (A, B) GBM2 conditioned media (CM) were prepared by incubating cells with medium alone (Ctr), IL-1ra (1 µg/ml), IL-1α (10 ng/ml) or both, as indicated (right panel, B). Control cultures were exposed to fresh medium (Ctr medium) or recombinant IL-1α (left panel, A). Human fetal mixed neuronal cultures were exposed to CM and neurotoxicity was measured by trypan blue exclusion at 72 h post incubation. Arrows indicate the examples of dead (blue) cells. (C) Number of trypan blue+ cells are counted in each conditions and data are pooled from three independent experiments. *** p<0.001, **p<0.01, * p<0.005 Neurotoxic activity was induced by glioma secretome (activated or non-activated), as well as recombinant IL-1α. IL-1ra completed reversed neurotoxicity in glioma CM.

Data shown in Figures 1–7 are representative of results obtained in at least two independent experiments. Data shown in Figures 8 and 9 are pooled from at least three independent experiments. For multiple comparisons, one-way ANOVA with Bonferroni post test was performed. For comparison of two groups, Student’s t-test was used. P values <0.05 were considered significant. All statistics were run using the GraphPad Prism 6.0 software.

## Results

### Expression of interleukin-1 in human glioblastoma cells

#### Human glioma cells produce IL-1 mRNA and protein (Figure 1)

IL-1 expression by non-myeloid cells is considered highly unusual, but given the reports that certain systemic non-myeloid human cancer cells produce IL-1 [45], we investigated whether human malignant glioma cells produce IL-1. Several human GBM cell lines including U251 and U87 were stimulated with three major types of immune activators (LPS, poly IC and IL-1/IFNγ) that are commonly used to activate astrocytic cells *in vitro*. IL-1 expression was examined by real-time PCR (Q-PCR) and by ELISA which measures both pro- and mature-forms of IL-1β. Culture supernatants and cell lysates were separately analyzed for intracellular and extracellular (secreted) IL-1 proteins. The results are compared with those obtained in primary human fetal astrocytes and microglia.

Representative results are shown in Figure 1 (A: intracellular IL-1β, B: secreted IL-1β, and C: Q-PCR). U251 and U87 cells responded similarly. LPS had no effect, poly IC had little or no effect but IL-1 (with or without IFNγ) strongly induced IL-1 itself, confirming our previous observations that IL-1 but not LPS activates *human* astrocytic cells and that poly IC is a weak inducer of *pro*inflammatory cytokines in these cells [26], [27], [39], [46]. Interestingly, primary human astrocytes did not produce IL-1β protein, although they expressed large amounts of IL-1β mRNA following stimulation with IL-1α (Figure 1C) [39]. Human microglial IL-1 was induced by LPS >poly IC with IL-1 itself having a much weaker effect, consistent with the known response of myeloid lineage cells. The amounts of IL-1 protein produced in glioma cells were in the same order of magnitude as microglia (ng/ml intracellular and pg/ml secreted IL-1 with some variations) (see below). Data shown are IL-1β (mRNA and protein) expression using IL-1α as the cell activator. *Results were similar when IL-1α expression was examined using IL-1β as the cell activator (data not shown).* IL-1β mRNA levels determined by Q-PCR from three separate cases of astrocytes, U87 and U251 cells (control and IL-1/IFNγ-stimulated levels) are listed in Table S1.

#### IL-1 expression in patient-derived glioblastoma cells (Figure 2)

Three patient-derived glioma cell lines (one low grade astrocytoma and two GBM) were tested for their ability to produce IL-1 proteins. As shown in Figure 2, IL-1β protein production was observed in GBM cells but not in low grade astrocytoma cells (Figure 2A). In one GBM cells (GBM2), IL-1β was produced spontaneously (with variability, see below) and this was potentiated following stimulation with IL-1α (± IFNγ). In addition, a significant portion of IL-1β was secreted (also see below). The amount of IL-1β production was input IL-1α dose-dependent at 0.1 pg/ml - 10 ng/ml (Figure 2B), demonstrating that GBM cells are exquisitely sensitive to IL-1 stimulation. Immunostaining of the GBM2 monolayer showed cytosolic IL-1β immunoreactivity in a subset of untreated cells (Figure 2C). *Together, these results show the ability of human malignant glioma cells to produce IL-1 protein spontaneously and following stimulation with IL-1. Non-neoplastic (or low grade) human astrocytes do not produce IL-1 protein even when they express IL-1 mRNA*. *The ability to produce IL-1 protein appears to be common to all GBM cells tested.*

#### Inflammasome activation in GBM cells (Figure 3)

The cleavage and release of bioactive IL-1β in myeloid cells is mediated by the inflammasomes, cytosolic caspase-1 activating tri-molecular complexes consisting of pro-caspase-1, the adaptor protein ASC and a NLRP3 (or AIM) protein [47]. The most studied inflammasome is the NLRP3 inflammasome with ATP or nigericin as the trigger of NLRP3 activation. We therefore asked whether similar mechanisms operate in glioma cells. GBM2 and U251 cells were stimulated with IL-1α with or without ATP or nigericin and the amounts of IL-1β in the cell extract (intracellular) and supernatants (secreted) were analyzed by Western blot and ELISA (Figure 3) (Figure S1). Western blot results show that intracellular proIL-1β (∼32 kDa) as well as secreted mature IL-1β (17 kDa) were induced by IL-1 stimulation. Treatment with ATP or nigericin further increased the amount of 17 kDa mature IL-1β, while reducing the amount of 32 kDa proIL-1β. Results were similar in both GBM2 and U87 cells. Increased IL-1β secretion by ATP and nigericin was also demonstrated by ELISA. Furthermore, western blot analysis showed that NLRP3 protein expression was increased by ATP and nigericin. Immunoprecipitation experiments showed that NALP3 was complexed with ASC, the adaptor protein in the inflammasome, in both control and IL-1 induced conditions and the amount appeared to increase with ATP and nigericin. Treatment of cells with siRNA specific to NLRP3 significantly reduced the amount of secreted IL-1β in all conditions (Figure 3C and D). *Together, our results show that in GBM cells, IL-1 provides the mechanism by which IL-1 expression is induced (signal 1) as well as processed and secreted (signal 2). Signal 2 is activated by the NLRP3 inflammasome. Similar to myeloid cells, ATP and nigericin activate NLRP3 inflammasome in GBM cells.*

#### Role of annexin A2 in IL-1β production (Figure 4)

A2 is a pro-tumor gene expressed in malignant gliomas *in vivo* that is implicated in tumor invasiveness, proliferation and angiogenesis [48], [49]. Annexin A2 has also been shown to modulate macrophage activation as well [50], [51]. In murine dendritic cells challenged with particulate wear debris, A2 has been shown to stabilize endosomal membrane and inhibits inflammasome activation [52]. We therefore asked whether A2 was involved in IL-1 production in GBM cells. Figure 4 shows the results of these experiments. A2 protein was highly expressed in GBM cells compared to normal human astrocytes (Figure 4A). In both U251 and U87 cells, A2 protein knockdown by siRNA (Figure 4B) also suppressed IL-1 production (both intracellular and secreted) demonstrating a *positive* role A2 plays in IL-1 production in these cells (Figure 4C, D). *Together these results demonstrate that annexin A2 contributes positively to the generation of IL-1 (synthesis and secretion) in GBM cells. They also suggest that the known pro-tumor activity of A2 in GBM may in part be mediated by IL-1* (see Discussion).

#### IL-1 translation is actively suppressed in human astrocytes (Figure 5)

Our data suggest that while human astrocytes do not translate IL-1 mRNA to proteins, this translational block is completely lifted in GBM cells. This is a highly unusual and intriguing finding with no similar precedents. We began to explore the underlying mechanism first by asking whether human astrocytes have a mechanism that actively suppresses IL-1 expression. Human fetal astrocytes or HEK293 cells were transfected with an IL-1 expression plasmid (IL-1β/GFP) or a control GFP plasmid and GFP and IL-1β expression was examined 24 h later by fluorescence microscopy and western blot analysis, respectively (Figure 5). These experiments showed that while IL-1 and GFP (fusion protein) were readily expressed in HEK293 cells transfected with the IL-1β/GFP plasmid, neither was expressed in human astrocytes. By contrast, GFP was readily expressed when cells were transfected with the GFP plasmid. *These results strongly suggest the presence of a suppressive mechanism that blocks the expression of IL-1 protein in human astrocytes.*

#### Role of miR-132/121 in human astrocyte IL-1 synthesis (Figure 6)

We next asked whether microRNAs play a role in the translational block of IL-1 in human astrocytes. Of the several microRNAs implicated in translational suppression of human cells, we chose miR-132/212 based on our target prediction search (http://www.microrna.org/microrna/home.do) which identified miR-132/212 being able to target both IL-1α and IL-1β [IL-1α: miR-330-5p, 326, 211, 204, 488, 185, 328, 149, 299-3p, 495, 590-5p, 21, *132, 212*, 340 and 144; IL-1β: miR-505, 200a, 141, 30e, a, d, c, b, 543, 181d, b, c, a, *212, 132*, 24, 543, 448, 203, 136, 205, 410, 340, 374a, b, 365, 874, 150, 149, 125a-3p, 590-3p, 140-5p, 494, 194 and 653]. Alignment of miR-212, miR-132 and IL-1α, IL-1β sequences is provided in Table S2. These target sequences are localized in the IL-1 coding sequences and are also present in the plasmid used in the overexpression experiments in Figure 5. miR-132 and miR-212 have similar mature sequences, share the same seed region, apparently target the same mRNAs, and are involved in the development and function of neurons and immune cells [53]. We tested the effects of miR-132 and miR-212 inhibitors to determine whether the IL-1 translational block in astrocytes can be released by them. But despite the effective suppression of miR-132 by both inhibitors (A), there was no change in the amount of IL-1β produced human astrocytes following transfection with the anti-miRs (B). *These results exclude a role for miR-132/121 in the suppression of IL-1 expression in human astrocytes*. To exclude the possibility that the lack of IL-1β protein expression is due to rapid protein degradation rather than translational block, we tested the effect of a proteasome inhibitor, lactacystin. As shown in Figure 6C, lactacystin had no effect on astrocyte IL-1β expression. *These results together demonstrate that absence of IL-1 protein expression in human astrocytes is most likely due to active repression of translation*.

### Role of interleukin-1 in glioma progression

In the next few experiments, we directly explored the role of IL-1 in GBM cell signal transduction, angiogenesis and neurotoxicity in our *in vitro* models to determine how IL-1 might participate in glioma progression and CNS pathogenesis.

#### IL-1 activates Stat3 in glioma cells (Figure 7)

Stat3 is a transcription factor central to cancer progression including in gliomas [31], [32]. Stat3 is constitutively activated in a subset of malignant gliomas and the expression of constitutively active Stat3 (together with C/EBPβ) has been shown to transform gliomas to a more aggressive mesenchymal subtype [54]. Stat3 is typically activated by the IL-6 family cytokines/growth factors as well as by the Th2 cytokine IL-10 [32], [34], but whether it can be activated by IL-1 is not known. We stimulated U251 and U87 cells with IL-1β (± IFNγ) and the amounts of activated (pStat3) and total Stat3 were determined at different time points by western blot (Figure 7A, B). The results were similar with or without IFNγ. In both U87 and U251, increased pStat3 was detected at 30 min and 60 min but not 360 min, showing that IL-1 (±IFN) transiently increased the amount of pStat3 in GBM cells. *The results suggest that chronic IL-1 production by glioma can cause sustained Stat3 activation.*

#### IL-1-induced U87 secretome promotes angiogenesis in vitro (Figure 8)

In order to test whether IL-1 promotes angiogenesis we performed a tube formation assay using HUVEC as the source of endothelial cells and IL-1-stimulated U87 cell conditioned medium (CM) as the incubation medium. The BD BioCoat Angiogenesis System-Endothelial Cells Tube Formation Matrigel Matrix 96-well plate was used to measure tube formation. U87 glioma CM was prepared by treating cultures with medium alone, IL-1, or IL-1 plus IL-1ra, followed by extensive washing of cells and further incubation with fresh medium. HUVEC cells suspended in U87 CM were added to Matrigel Matrix 96-well plates and the number of closed networks (vessel-like tube formation) was determined after 18 h. The results are shown in Figure 8. The presence of IL-1 in the stimulating medium increased the number of closed network (∼3-fold) and this was completely reversed by co-incubation with IL-1ra. *These results show that IL-1-induced glioma secretomes contain factors that promote tube formation (tumor angiogenesis), supporting the conclusion of our recent proteomics study* [14].

#### Glioma releases neurotoxic substances in response to IL-1 (Figure 9)

To examine whether IL-1 treated GBM cells secrete neurotoxic substances, glioma CM were prepared as described for the tube formation assay above. Neurotoxic activity of the glioma secretome was determined in mixed human fetal CNS cell cultures containing neurons, as previously described [22], [25], [26]. Fresh (Ctr) medium and recombinant IL-1α was used as negative and positive controls for neurotoxicity assays, respectively. Neurotoxicity was determined by viral dye (trypan blue) exclusion 72 after incubation with glioma CM. Figure 9 shows data obtained using GBM2 cell CM. Both the control and IL-1-activated glioma secretome contained neurotoxic activity (IL-1> Ctr) and the presence of IL-1ra in the incubation medium reduced the neurotoxic activity in both CM. Similar results were obtained with U251 cells pre-treated with IL-1/IFNγ (±IL-1ra) (Figure S2). *These results suggest that GBM secretomes in vivo can induce neurotoxicity and that this can be potentiated by the presence of IL-1 in the glioma microenvironment.*

## Discussion

Our study shows the novel finding that *IL-1 expression is aberrantly induced in malignant glioma cells.* The ability of various astroglioma cells to spontaneously produce low picogram/ml of IL-1 *in vitro*, as well as IL-1 expression by malignant glioma cells *in vivo* have been reported [55]–[57]. Our study in part confirms that GBM cells can spontaneously produce IL-1β or show IL-1 activity (Figures 2A, C and Figure 9), though this was a highly variable finding. The unexpected and consistent finding was that human GBM cells produce IL-1 when activated (with IL-1) in amounts comparable to activated macrophages. Our results are significant in light of the fact that in human astrocytes, IL-1 expression is completely repressed at the translational step. Our results suggest the presence of active repressive mechanisms, as IL-1 expression was also undetectable in human astrocytes transfected with the IL-1 expression plasmid. We believe that micro-RNA-based suppression mechanism is unlikely, as inhibition of miR-132/212 (only microRNAs that can inhibit both IL-1α and IL-1β based on target prediction) had no effect on astrocyte IL-1 production. Involvement of other mechanisms such as miR-146a (an endogenous feedback inhibitor of cytokine expression including in astrocytes) [58], [59] is unlikely given the specific repression of IL-1 and not other cytokines. Failure of *human* astrocytes to produce IL-1 is a highly unusual and significant species-dependent neuroimmune mechanism, akin to the inability of human macrophages and microglia to express iNOS [60]–[62]. We propose that acquiring the ability to express IL-1 (a myeloid-specific protein) could induce epithelial-mesenchymal transition (EMT)-like transformation of glioma cells resulting in increased migratory capacity, a unique gene signature and increased immune signaling (NF-κB, pSTAT3).

The IL-1 system consists of two agonists IL-1α and IL-1β, the receptor antagonist (IL-1ra), the signaling receptor IL-1RI, as well as other newly discovered members [63]–[67]. Unlike other cytokines, IL-1β biogenesis requires two signals. Signal 1 activates the transcriptional and translational induction of proIL-1β (32 kDa). Signal 2 activates the proteolytic cleavage of proIL-1β to active IL-1β (17 kDa). Signal 2 involves activation of the cytosolic trimolecular complex (inflammasome) consisting of procaspase-1, adaptor protein ASC and a Nod-like receptor (NLR). The most studied NLR is NLRP3 as it senses a diverse type of stimuli and plays a significant role in many inflammatory diseases [16], [68]–[70]. Very little is known about the mechanism of inflammasome activation in non-myeloid cells. In keratinocytes, NLRP3 inflammasome is activated by UV irradiation [71], [72]. In human astrocytes (study limited to cells derived from a single donor) IL-1β production/secretion was induced by ATP (alone) *via* NLRP2 inflammasome [73]. The latter finding is highly unusual as ATP does not provide signal 1 in most cells. Furthermore, we do not detect IL-1β production in human astrocytes.

Our results in GBM cells showed that stimulation with IL-1 increased the amount of IL-1 mRNA and protein, as well as IL-1 secretion. Interestingly, we detect similar levels of NLRP3 protein (and the NLRP3-ASC complexes) before and after IL-1 stimulation, which suggested that inflammasome is constitutively active in GBM cells. In macrophages, baseline NLRP3 protein expression is minimal and upregulation of NLRP3 is the (possibly only) critical step involved in inflammasome activation [43], [74]. Consistent with this idea, treatment of GBM cells with ATP or nigericin resulted in both increased expression of NLRP3 and inflammasome activation (increased IL-1β release). Our finding that annexin A2, a molecule strongly implicated in glioma progression by many studies [48], [49], [75], has a critical positive role in glioma IL-1 synthesis is also novel. It is noteworthy that (soluble) A2 also has been shown to trigger cytokine (such as IL-1) synthesis in macrophages through TLR4 [50]. Together, these results show the resemblance between macrophages and GBM cells in the mechanism of IL-1 production. Given the highly overlapping nature of IL-1 and A2 in their activities associated with glioma progression [14], [75]–[77], it is possible that some of the A2 activities may in fact be mediated through IL-1.

Our studies of human and mouse astrocytes and glioma cells demonstrate that IL-1α mRNA and protein are co-induced and co-regulated with IL-1β, as in myeloid cells (data not shown). They also bind to the same receptor and have similar potency. However, activation of IL-1α does not require signal 2 (proIL-1α is bioactive), indicating that different mechanisms are involved in the activation/release of IL-1α and IL-1β. For example, tumor cell necrosis (an essential histologic criterion for GBM diagnosis) [12], [78] would be sufficient to release proIL-1α to the extracellular space. ATP is also released from dying cells likely contributing to inflammasome activation. Many of the downstream events in IL-1-activated cells resemble those exposed to hypoxia including activation of HIF-1α [79], [80]. These findings suggest that the biological sequelae of tumor necrosis are linked to those of IL-1 activation.

Our results also show that GBM cells are highly sensitive to IL-1 stimulation (0.1 pg/ml triggered IL-1β production), representing ∼two orders of magnitude higher sensitivity than human astrocytes (data not shown). These results suggest that IL-1-induced modulation of glioma cells is likely physiologically relevant. It is also important to note that the critical steps of IL-1 regulation in human CNS are likely to be at multiple levels, as shown by discordant mRNA and protein expression by human astrocytes, as well as the presence of IL-1α/β activation/release mechanisms that are not under transcriptional control. The latter shows the limitation of relying on The Cancer Genome Atlas (TCGA) as the principal guide to understanding of the complex tumor biology. We further entertain the possibility that aberrant IL-1 expression may be limited to a certain glioma subtype. Recent advances in cancer genomics/genetics have shown molecular subclassification of GBM that correlates with prognosis [81], [82]. We propose that IL-1 expression may be seen in the mesenchymal subtype characterized by high degree of inflammation and tumor necrosis. We further hypothesize that the IL-1 expressing gliomas might be separate from those with EGFR mutation/amplifications [12], [83]. These studies are important in understanding of the heterogeneity of glioma biology and have practical implications for personalized (or precision) therapy. Furthermore, detection of IL-1 protein in astrocytic cells by immunohistochemistry could be useful as a tumor biomarker. If future studies identify the specific mechanism by which IL-1 expression is repressed in astrocytes, this could provide new therapy targets. A broad anti-IL-1 therapy can also be contemplated as there are three FDA-approved anti-IL-1 agents with few side effects: IL-1ra (anakinra), a neutralizing mAb to IL-1β (canakinumab), and a soluble IL-1R (rilonacept). Lastly, the effect of IL-1 on glioma secretome promoting neuronal killing in our study suggests that glioma can compromise the integrity of the brain tissue and CNS function. IL-1 has additional deleterious effects such as induction of seizure activity [84], [85] and suppression of neuronal growth factor production from microglia [25], [86], indicating additional pathways glioma-induced IL-1 can negatively impact CNS function and quality of patients’ lives [87].

## Supporting Information

Figure S1 **Inflammasome activation and IL-1β secretion in GBM cells.** Average densitometry data from two independent experiments are shown for GBM2 and U87 cells. Experiments are performed as described in Figure 3 legend.
(TIF)

Figure S2 **U251 secretome neurotoxicity assay.** U251 cells were stimulated with IL-1+IFNγ and the neurotoxicity assay was performed as described in Figure 9. Mixed primary human fetal neuronal glial cultures were stimulated with medium alone (Ctr = 1), IL-1β + IFNγ ( = 2), conditioned medium from IL-1β/IFNγ-stimulated U251 cells prepared as described ( = 3) or conditioned medium from U251 cells stimulated with IL-1β/IFNγ + IL-1ra as described ( = 4). (A) Representative photographs of Trypan blue assay. (B) Number of dead neurons in four different conditions (1–4) in triplicate cultures (mean ± SD). *** p<0.001.
(TIF)

Table S1 **Relative IL-1β mRNA expression by primary human astrocytes and GBM cells.** Q-PCR was performed as described in the Methods section using PBDA or GAPDH as endogenous controls. The median value of the replicates for each sample was calculated and expressed as the cycle threshold (*C_T_*; cycle number at which each PCR reaches a predetermined fluorescence threshold, set within the linear range of all reactions). Δ*C_T_* was calculated as *C_T_* of endogenous control gene minus *C_T_* of target gene in each sample. The relative amount of target gene expression in each sample was then calculated as 2^Δ*CT*^. Data represent values from three different cases of human fetal astrocyte cultures, as well as three separate preparations of U87 and U251 cells. Unstimulated (Ctr) or stimulated with IL-1/IFNγ for 6 h.
(DOCX)

Table S2 **Alignment of sequences (miR-212, miR-132, IL-1α and IL-1β).**
(DOCX)
