# Supplementary figures and images for: Aberrant Expression of Interleukin-1β and Inflammasome Activation in Human Malignant Gliomas
Source: PLoS One. 2014 Jul 23;9(7):e103432. doi: 10.1371/journal.pone.0103432 (PMC4108401; doi:10.1371/journal.pone.0103432)

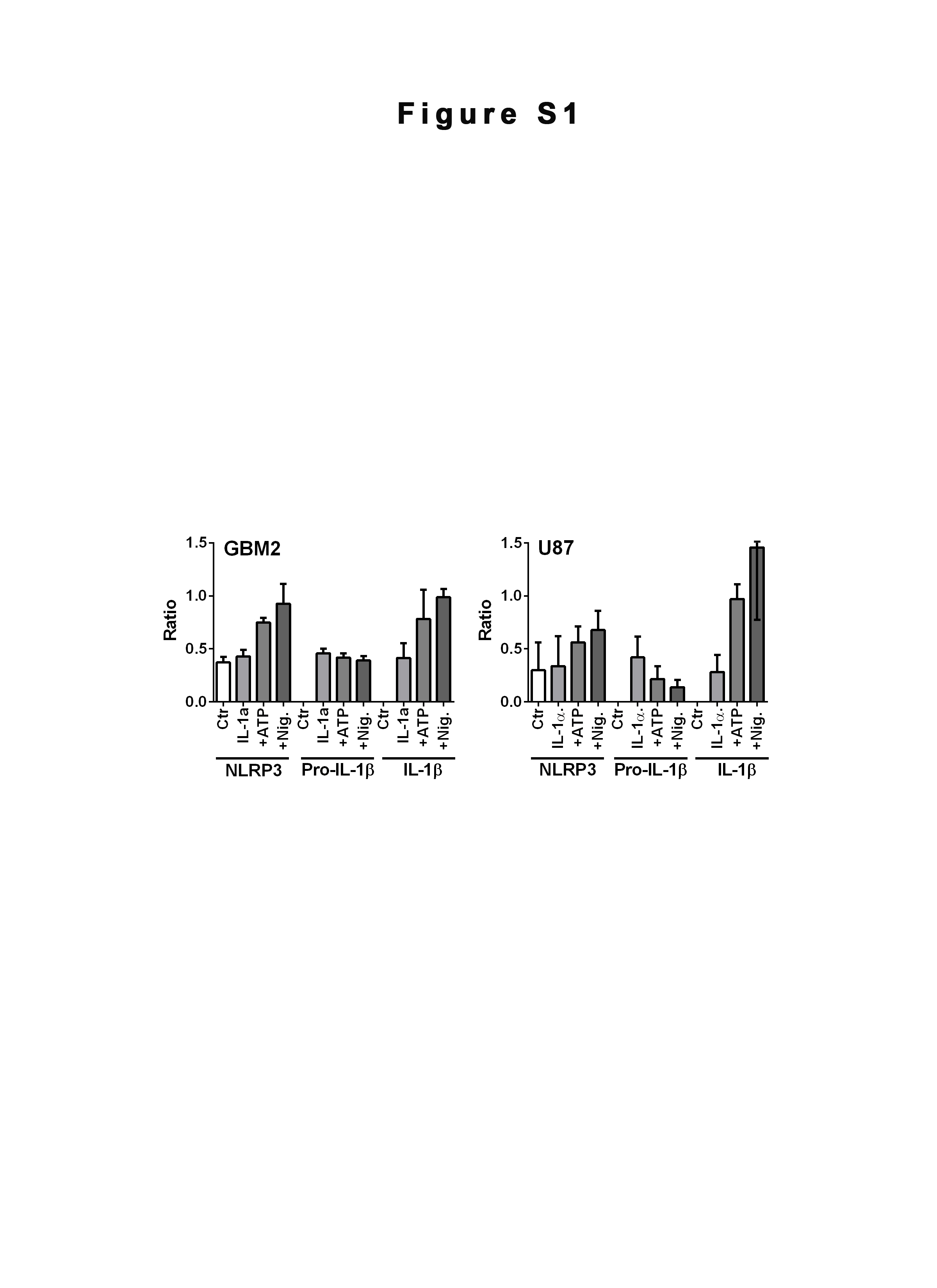

Supplement: Figure S1 — Inflammasome activation and IL-1β secretion in GBM cells. Average densitometry data from two independent experiments are shown for GBM2 and U87 cells. Experiments are performed as described in Figure 3 legend. (TIF) [file pone.0103432.s001.tif]

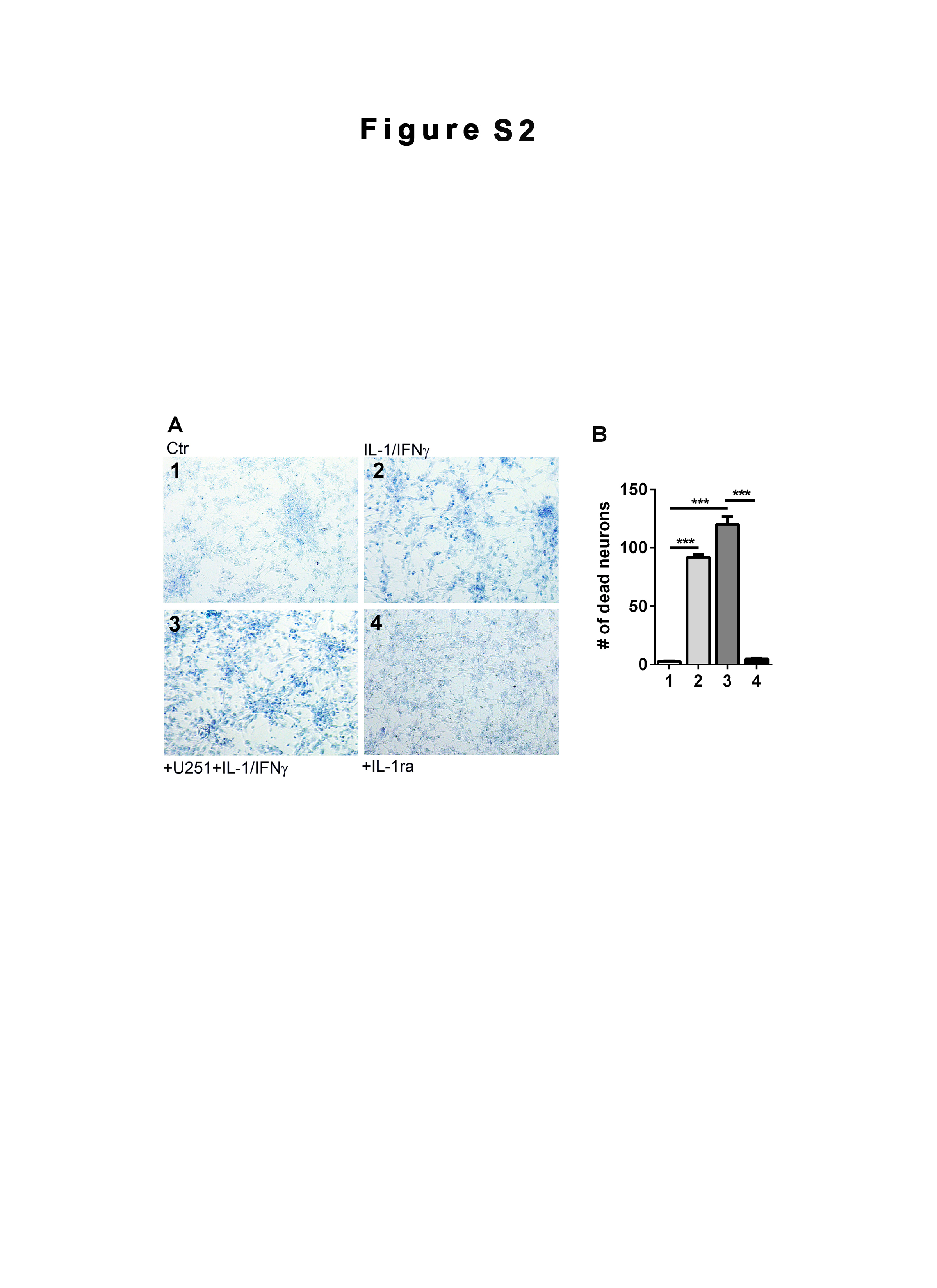

Supplement: Figure S2 — U251 secretome neurotoxicity assay. U251 cells were stimulated with IL-1+IFNγ and the neurotoxicity assay was performed as described in Figure 9. Mixed primary human fetal neuronal glial cultures were stimulated with medium alone (Ctr = 1), IL-1β + IFNγ ( = 2), conditioned medium from IL-1β/IFNγ-stimulated U251 cells prepared as described ( = 3) or conditioned medium from U251 cells stimulated with IL-1β/IFNγ + IL-1ra as described ( = 4). (A) Representative photographs of Trypan blue assay. (B) Number of dead neurons in four different conditions (1–4) in triplicate cultures (mean ± SD). *** p<0.001. (TIF) [file pone.0103432.s002.tif]
