# Supplementary material for: Aberrant Expression of Interleukin-1β and Inflammasome Activation in Human Malignant Gliomas
Source: PLoS One. 2014 Jul 23;9(7):e103432. doi: 10.1371/journal.pone.0103432 (PMC4108401; doi:10.1371/journal.pone.0103432)
Supplement: Table S1 — Relative IL-1β mRNA expression by primary human astrocytes and GBM cells. Q-PCR was performed as described in the Methods section using PBDA or GAPDH as endogenous controls. The median value of the replicates for each sample was calculated and expressed as the cycle threshold (CT; cycle number at which each PCR reaches a predetermined fluorescence threshold, set within the linear range of all reactions). ΔCT was calculated as CT of endogenous control gene minus CT of target gene in each sample. The relative amount of target gene expression in each sample was then calculated as 2ΔCT. Data represent values from three different cases of human fetal astrocyte cultures, as well as three separate preparations of U87 and U251 cells. Unstimulated (Ctr) or stimulated with IL-1/IFNγ for 6 h. (DOCX) [file pone.0103432.s003.docx]

| **Table S1.** | Relative level of IL-1β mRNA by Q-PCR (2^∆Cτ^) | | |
| --- | --- | --- | --- |
|  |  |  |  |
|  | **Ctr** | **IL-1/IFNγ** |  |
| **Astrocytes** | 0.022598 | 19.789616 |  |
|  | 0.028484 | 22.536453 |  |
|  | 0.026437 | 20.427212 |  |
| **U87** | 0.193451 | 15.019224 |  |
|  | 0.172115 | 24.571788 |  |
|  | 0.156471 | 19.980545 |  |
| **U251** | 0.045281 | 149.374869 |  |
|  | 0.056632 | 198.456182 |  |
|  | 0.036514 | 142.578904 |  |
|  |  |  |  |
