# Supplementary material for: Aberrant Expression of Interleukin-1β and Inflammasome Activation in Human Malignant Gliomas
Source: PLoS One. 2014 Jul 23;9(7):e103432. doi: 10.1371/journal.pone.0103432 (PMC4108401; doi:10.1371/journal.pone.0103432)
Supplement: Table S2 — Alignment of sequences (miR-212, miR-132, IL-1α and IL-1β). (DOCX) [file pone.0103432.s004.docx]

**Table S2.** Alignment of sequences (miR-212, miR-132, IL-1α and IL-1β)

**hsa-miR-212/IL1A Alignment**

| 3' ccggcacugaccucUGACAAu 5' hsa-miR-212  \|\|\|\|\|\|   65:5' aagcuaaauccuuuACUGUUa 3' IL1A | \|  \|  \| \| --- \| --- \| \|  \|  \| |
| --- | --- | --- | --- | --- | --- |

**hsa-miR-132/IL1A Alignment**

| 3' gcugguaccgacaucUGACAAu 5' hsa-miR-132  \|\|\|\|\|\|   64:5' gaagcuaaauccuuuACUGUUa 3' IL1A | \|  \|  \| \| --- \| --- \| \|  \|  \| |
| --- | --- | --- | --- | --- | --- |

**hsa-miR-212/IL1B Alignment**

| 3' ccggcacugaccucUGACAAu 5' hsa-miR-212  \|\|\|\|\|\|   553:5' ugagcaaauaucauACUGUUc 3' IL1B | \|  \|  \| \| --- \| --- \| \|  \|  \| |
| --- | --- | --- | --- | --- | --- |

**hsa-miR-132/IL1B Alignment**

| 3' gcugguaccgacaucUGACAAu 5' hsa-miR-132  \|\|\|\|\|\|   552:5' augagcaaauaucauACUGUUc 3' IL1B | \|  \|  \| \| --- \| --- \| \|  \|  \| \|  \| \| |
| --- | --- | --- | --- | --- | --- | --- | --- |
